# Sialolith of Unusual Size and Shape in the Anterior Segment of the Submandibular Duct

**DOI:** 10.7759/cureus.24114

**Published:** 2022-04-13

**Authors:** Georges Aoun, Carlo Maksoud

**Affiliations:** 1 Oral Medicine and Maxillofacial Radiology, Lebanese University, Beirut, LBN

**Keywords:** unusual size, unusual shape, submandibular duct, submandibular gland, sialolith, sialolithiasis

## Abstract

Sialolithiasis is a common condition characterized by the formation of salivary stones or calculi, also known as sialoliths, within a salivary gland or its duct. Usually, sialolithiasis presents as salivary gland swelling with intermittent pain around mealtime. All salivary glands can develop sialolithiasis, yet it occurs most commonly in the submandibular gland. In this report, we describe an unusual large sialolith measuring 1.7 cm and uncommonly resembling a canine tooth. The sialolith was removed surgically via intraoral approach, and the salivary secretion was restored.

## Introduction

Sialolithiasis is a frequent salivary gland condition in which calcifications or calculi (sialoliths) form inside salivary glands or in their ductal system [1]. It is estimated to occur in 1.2% of the adult population with a slight male predominance [2,3]. Sialoliths are usually unilateral and localized within the duct more frequently than inside the gland [4,5]. Their size is variable, ranging from a few millimeters to several centimeters. Giant or unusually large sialoliths are referred to those whose dimension exceeds 1.5 cm [6-8]. Any salivary gland can develop sialoliths, with the most common being submandibular (80%-95%) followed by the parotid (5%-20%) glands [2,3,7,9]. The sublingual and minor salivary glands are rarely affected (1%-2%) [9]. The submandibular gland is the most involved site because of its retrograde anatomical location and tortuous duct combined with the secretion of mucous and alkaline saliva [8,10]. In this report, we describe a case of a sialolith of unusual size and shape located in the submandibular duct.

## Case presentation

**Figure 1:**
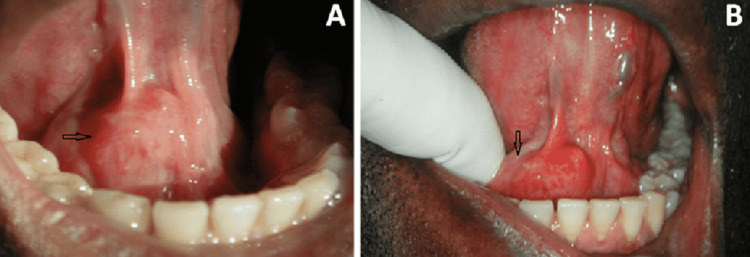
Intraoral photographs (A) The swelling under the patient’s tongue (black arrow); (B) digital palpation of the swelling (black arrow).

A 34-year-old male patient presented to our oral and maxillofacial surgery center complaining of swelling under his tongue (Figure 1A). Digital palpation revealed a stony hard, mobile, painless swelling on the right side of the floor of the mouth (Figure 1B).

**Figure 2:**
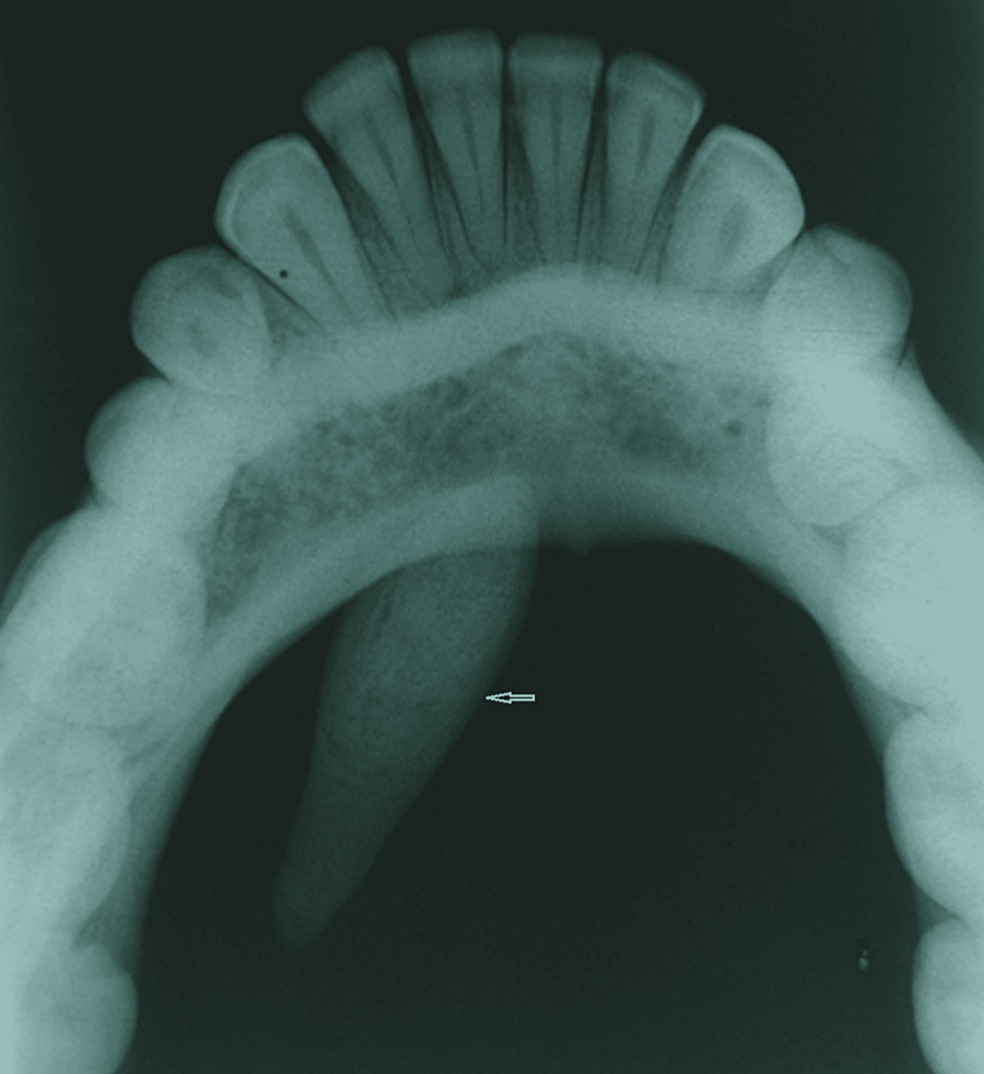
Topographic mandibular occlusal radiograph Topographic mandibular occlusal radiograph showing an unusually large sialolith (white arrow) on the right side of the floor of the mouth.

The overlying mucosa was stretched and shiny with reddish area on the superior aspect of the swelling. Salivary flow was negligible from the right submandibular duct orifice. On palpation, no regional lymphadenopathy was noticed. The presence of an unusually large sialolith in the region of the submandibular duct was confirmed by a topographic mandibular occlusal radiograph that showed a radiopaque mass resembling a canine tooth, anteriorly projected under the right inferior border of the mandible close to the genial tubercle (Figure 2).

**Figure 3:**
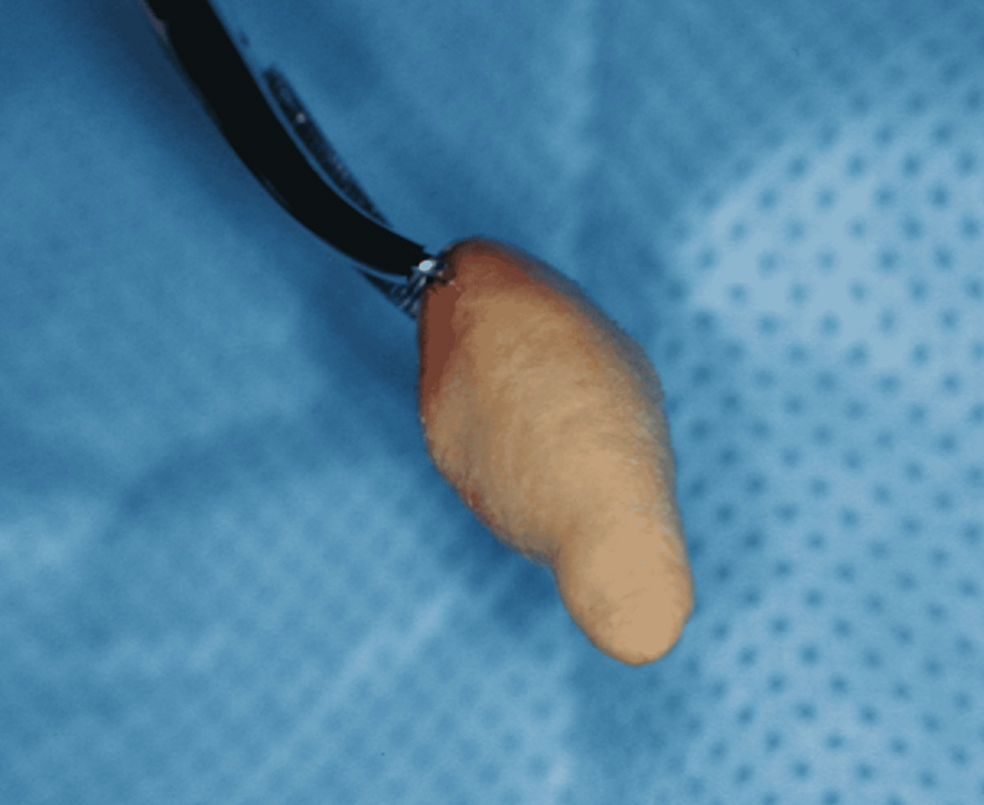
Photograph of the removed sialolith Photograph showing the size and shape of the removed sialolith.

The sialolith was removed surgically via intraoral approach under local anesthesia. The procedure consisted in enlarging the ductal orifice with an incision of 10 mm approximately after fixing the sialolith by a distal suture. A gentle pressure was then applied distally provoking the release of the sialolith through the incision. After that, an everted suture of the incised mucosa was performed, thus creating a salivary fistula. The sialolith removed was lightweight and presented many porosities. Its shape was unusually similar to a canine tooth and measured 1.7 cm in length (Figure 3).

No postoperative complications were noted. Salivary flow was restored after the removal of the sialolith.

## Discussion

The submandibular gland is the second largest of the three major salivary glands, the others being the parotid and sublingual glands. It lies in the submandibular triangle and presents two lobes, superficial and deep, separated by the mylohyoid muscle [11]. The submandibular duct arises from the deep lobe of the gland and emerges anterosuperiorly and medially to finally drain in a narrow orifice at the sublingual caruncle located lateral to each side of the base of the frenulum linguae [12]. The submandibular gland is responsible for approximately 70% of unstimulated saliva.

The exact pathogenesis of sialolithiasis remains unclear. Some believe that sialoliths result from the deposition of mineral salts on nidi of bacteria, mucus, or desquamated cells [3,13]. The submandibular gland is most affected by sialolithiasis because of its saliva composition (higher concentration of calcium and phosphate and higher mucous levels) and alkaline pH [3]. In addition, the long and tortuous path of the submandibular duct results in a flow against gravity that facilitates saliva stasis. This stasis produces a change in the mucoid element of saliva, therefore forming a gel that constitutes the framework for the deposition of salts and organic substances resulting in the formation of calculi [14].

Sialoliths are yellowish, round or oval masses and can be rough or smooth [9]. They usually measure less than 1 cm and rarely more than 1.5 cm where they are considered unusually large or giant [6,8]. These latter have been mostly reported in the glands than in the ducts [3]. Sialoliths may be asymptomatic; approximately 30% of the cases of submandibular sialolithiasis present with painless swelling. On the other hand, when symptomatic, pain and swelling of the involved gland during food intake are the most common clinical symptoms [3,8,9].

The differential diagnosis of sialolithiasis includes oral bacterial infections, Sjögren's syndrome, sarcoidosis, chronic sclerosing sialadenitis (Kuttner's tumor), and neoplastic or non-neoplastic lesions. Appropriate diagnosis depends on a thorough medical history along with clinical and radiological evaluations [15]. Radiographic techniques used in sialolithiasis diagnosis depend on the size of sialoliths. Large calculi can be identified by panoramic or occlusal radiographs, whereas smaller or hypomineralized ones need more sophisticated techniques such as sialography, scintigraphy, computed tomography (CT), magnetic resonance imaging (MRI), ultrasonography, and sialoendoscopy [9,16].

Sialography consists of radiography after injecting a contrast medium into the salivary duct. It helps visualizing the entire glandular system. This technique is contraindicated in patients allergic to contrast medium and suffering from acute infections. Moreover, sialography is not indicated in the case of sialolith suspected in the posterior segment of the duct which could be moved toward the body of the gland thus complicating its removal [17]. Scintigraphy and CT scan are less invasive techniques compared to sialography and can be successfully used when the latter is contraindicated [9]. On the other hand, ultrasonography that uses ultrasound waves of frequency 20K Hz or higher to identify sialoliths is very accurate except for calculi < 2 mm where the lack of acoustic shadows may lead to diagnostic mistakes [7]; nevertheless, ultrasonography of the maxillofacial complex is operator dependent and needs to be performed by an experienced clinician, otherwise other modalities are recommended (such as CT scan). Sialoendoscopy, a recently developed diagnostic and interventional technique, allows direct visualization of the salivary duct lumen (presence of sialoliths, polyps, foreign body, etc.) using a fiberoptic endoscope [4].

The most suitable treatment is selected according to the size and location of the sialolith [6]. Small calculi may often be released by “milking” the duct and using bimanual palpation, while giant accessible calculi are surgically removed. Removal of an entire lobe or gland is reserved for the cases where the calculi are deeply embedded in the salivary gland parenchyma [18].

In this report, a sialolith of unusual size (1.7 cm long) and shape (resembling a canine tooth) was presented. Very few cases of sialolith mimicking a canine tooth have been reported in the literature. Additionally, sialoliths of this size are usually symptomatic and rarely located in the salivary duct; in our case, the sialolith was located in the anterior segment of the duct and was completely asymptomatic. A topographic mandibular occlusal radiograph was sufficient to confirm its presence, and the appropriate surgical treatment has restored salivary secretion.

## Conclusions

Sialolithiasis is one of the most common salivary gland pathologies, usually manifesting as swelling with intermittent pain around meals. Hence, painless sialoliths of unusual size and shape located in the submandibular duct are rare. Careful clinical and radiological assessments are crucial for establishing the appropriate diagnosis and treatment. The management of sialolithiasis is based on the size and location of the sialolith. While accessible unusually large calculi may be surgically removed, inaccessible ones require the removal of an entire lobe or the whole gland.
